# A Study to Assess the Prevalence and Determinants of Compulsive Buying Disorder Among College Students in Chengalpattu District, Tamil Nadu

**DOI:** 10.7759/cureus.85664

**Published:** 2025-06-09

**Authors:** Nikhil CM, Shanthi Edward, Angeline Grace, Swetha NB

**Affiliations:** 1 Community Medicine, Sree Balaji Medical College and Hospital, Chennai, IND

**Keywords:** compulsive behaviors, impulsive behaviors, medical students, mental health, online shopping behavior

## Abstract

Introduction

Compulsive buying disorder (CBD), also referred to as compulsive buying-shopping disorder, shopping addiction, oniomania, or pathological buying, is characterized by excessive and poorly controlled preoccupations, urges, or behaviors related to shopping and spending. Digitalization in e-commerce and payment systems has made indulgence in shopping easier than ever. CBD is a growing concern, especially in the younger generation.

Aim

To determine the prevalence of CBD and its associated factors among undergraduate medical students.

Methodology

A cross-sectional study was done among undergraduate medical students in a tertiary college in Chengalpattu district, Tamil Nadu, India. A total of 300 students participated in the study. A pretested semi-structured questionnaire was used to collect socio-demographic details and information on factors associated with CBD. A validated scale was used to assess CBD. Data analysis was done using IBM SPSS Statistics for Windows, Version 25 (IBM Corp., Armonk, NY).

Results

The prevalence of CBD was found to be 68/300 (22.6%) among the undergraduate medical students. A total of 26/103 (25.2%) males were affected compared to 42/197 (21.3%) female students. Being tempted to buy during deals and offers, social media influence, and not having a habit of saving money were found to be significantly associated with CBD.

Conclusion

CBD is an often-overlooked behavioral concern in academic settings, and its presence is highlighted in this study. These findings underscore the influence of social media, peers, and strategic deals by e-commerce services on students’ purchasing habits. Interventions to identify and treat CBD in the early stages are important to prevent long-term financial and psychological consequences.

## Introduction

Compulsive buying disorder (CBD), otherwise known as compulsive buying-shopping disorder, shopping addiction, oniomania, and pathological buying, is categorized as too much or perhaps improperly controlled obsessions, desires, or actions regarding going shopping and shelling out, despite not requiring or using those products, resulting in severe negative consequences for affected persons and their family and impairments in important areas of functioning [1,2].

We are in an era of technological innovation leading to rapid urbanization, which is accompanied by an increase in people’s average purchasing power. Once people reach a level of financial independence, they tend to develop shopping habits and indulge in buying things that are beyond their essential and daily needs. This condition becomes pathological when it affects their financial stability and affects their personal and professional life [3]. This condition is often rooted in pre-existing psychiatric conditions such as depression, stress, and anxiety. Shopping has become the only way to cope with stress. It has also been postulated that compulsive buying is associated with attention deficit hyperactivity disorder (ADHD) traits, self-rated depression, and lifetime psychological comorbidities [4].

It has been found that at least one-quarter of the global population indulges in compulsive buying at some point in their lifetime [5]. The common age group is 18 to 24 years of age, during which various factors such as self-esteem and social image gain importance in the lives of young adults [6]. The female gender is commonly involved in compulsive buying when compared with males, as women often tend to accept the fact that they enjoy shopping [7].

Schlosser et al. hypothesized that CBD occurs in four phases: anticipation, preparation, shopping, and spending [8]. The first phase begins when a person anticipates having a particular product he wishes to have and creates scenarios in his mind in which he is happy and content with that product. The second phase begins when an individual receives sufficient money to buy the product. In this phase, the person actively searches the Internet for the best available price and decides when and where to purchase the product. In the third phase, the act of shopping comes into play, in which a person visits a particular store and buys a product about which they are intensely excited [8]. The final act of purchase is usually followed by disappointment or letdown and guilt [9]. The motives or factors that predispose a person to involvement in CBD may include depression, anger, boredom, and anxiety, which are usually relieved after the completion of the act of buying things [10].

Although India is a developing country, there has been a rapid rise in e-commerce, leading to the availability of many online retailers selling all types of consumer items from fashion and jewelry to all types of electronics. Everything is digitally available, accompanied by a review system that provides a review of products. Therefore, a person with a CBD tends to obsessively scroll various webpages regarding the product and read a countless number of reviews before deciding [11]. A single critical negative review may leave them off, and a vicious cycle continues for the next product. This, in addition to the consequences of CBD, also increases screen time and various side effects associated with prolonged gadget use, such as headaches, anxiety, and depression [12].

Based on the above background, this study was conducted among medical students in a tertiary medical college and hospital to determine the prevalence of compulsive buying disorders and the associated factors that would have predisposed them to the condition.

## Materials and methods

Definition

CBD is categorized as improperly controlled obsessions, desires, or actions regarding going shopping and shelling out, despite not requiring or using those products, resulting in severe negative consequences for affected persons and their family and impairments in important areas of functioning.

Study setting

A cross-sectional study was done among undergraduate medical students at Sree Balaji Medical College and Hospital, a tertiary medical college situated in Chengalpattu district, Tamil Nadu, India. The total duration of the study was six months, from January 2024 to June 2024. Ethical approval was obtained from the Institutional Human Ethics Committee, Sree Balaji Medical College and Hospital (002/SBMCH/IHEC/2023/2095).

Sample size calculation

Since there is no previous study done on CBD in India, prevalence (P) was taken as 50% and applying the formula Z2 PQ/L2 with Z = 1.96, 95% Confidence Interval, P = 50%, Q = 50%, and absolute precision L = 6%, the minimum required sample size was calculated to be 267. Assuming a nonresponse rate of 10%, the sample size is rounded off to 300, which is the minimum required sample size.

Sampling method

The total number of undergraduate medical students in the selected medical college was 1250, who were distributed among regular and supplementary batches across four academic years. The participants were selected from all academic years using probability-proportional-to-size (PPS) sampling, which was used to determine the number of participants from each academic year. The participants from each year were line-listed and systematic random sampling was used to select participants from each year. In each batch, the first participant was chosen among the first five students in the line list by lottery method and subsequently, every fourth student was chosen. If a participant did not consent to participate in the study, the next person in line was approached to provide consent.

Data analysis

Data were entered into Microsoft Excel 2013 (Microsoft Corp., Redmond, WA, USA), and statistical analysis was performed using IBM SPSS Statistics for Windows, Version 25 (IBM Corp., Armonk, NY). Descriptive statistics are presented as tables and graphs. Bivariate analysis was performed using the Chi-square test, and variables that were found to be statistically significant at 95% CI were included in the logistic regression model. The strength of the association between CBD and related factors was quantified using adjusted odds ratios.

Study tools

A pretested semi-structured questionnaire was used to collect socio-demographic details, and details regarding CBD-like modes of shopping, payment methods, etc. (see Appendix A). The Richmond Compulsive Buying Scale [13] was used to measure CBD prevalence (see Appendix B). It is a 6-item questionnaire in which responses were graded on a Likert scale of 1-7. When a person scores above 25, they are considered to have compulsive buying disorder [13,14].

## Results

The sociodemographic features and sources of money of the study participants are listed in Table 1. Most study participants belonged to the age group of 21-24 years, comprising 168 (56%) students. The majority of the study participants were female students (197 (65.7%)). Among the study participants, only 14 (4.7%) had their own sources of income, while 62 (20.7%) of the respondents received financial support from people other than their parents.

**Table 1:** Distribution of socio-demographic variables among the study participants (n=300)

Variables	Category	Frequency N= 300	Percentage (%)
Age	17-20	132	44
	21-24	168	56
Gender	Female	197	65.7
	Male	103	34.3
Year of study	1st and 2nd year	164	54.7
	3rd and 4th year	136	45.3
Residence	Living away from parents	139	46.3
	Living with parents	161	53.7
Own Income Source	Yes	14	4.7
	No	286	95.3
Pocket money per month	More than Rs. 5000	98	32.7
	Less than Rs. 5000	202	67.3
Financial support from people other than their parents	Supported by others	62	20.7
	Not supported by others	238	79.3

The financial habits of study participants are given in Table 2. Seventy (23.3%) of them were accountable to their parents for their expenditures. Among the participants, 149 (49.7%) had savings or recurrent deposit accounts. Two-thirds of participants (198 (66%)) reported that their spending habits were not influenced by their social circles. The majority of the participants (232 (77.3%)) didn’t have the habit of saving money.

**Table 2:** Distribution of financial practices among the study participants (n=300)

Variables	Category	Frequency N= 300	Percentage (%)
Accountability to parents	Not accountable	70	23.3
	Accountable	230	76.7
Has savings or recurrent deposit account	Doesn't have a savings account	149	49.7
	Has savings account	151	50.3
Tempted to buy during offers and deals	Yes	135	45
	No	165	55
Ever been scolded by parents for spending too much	Yes	117	39
	No	183	61
Spending influence by social circle	Yes	102	34
	No	198	66
Social media influence on spending	Yes	126	42
	No	174	58
Habit of saving money	Yes	68	22.7
	No	232	77.3
Been taught about financial saving	Yes	100	33.3
	No	200	66.7

**Figure 1:**
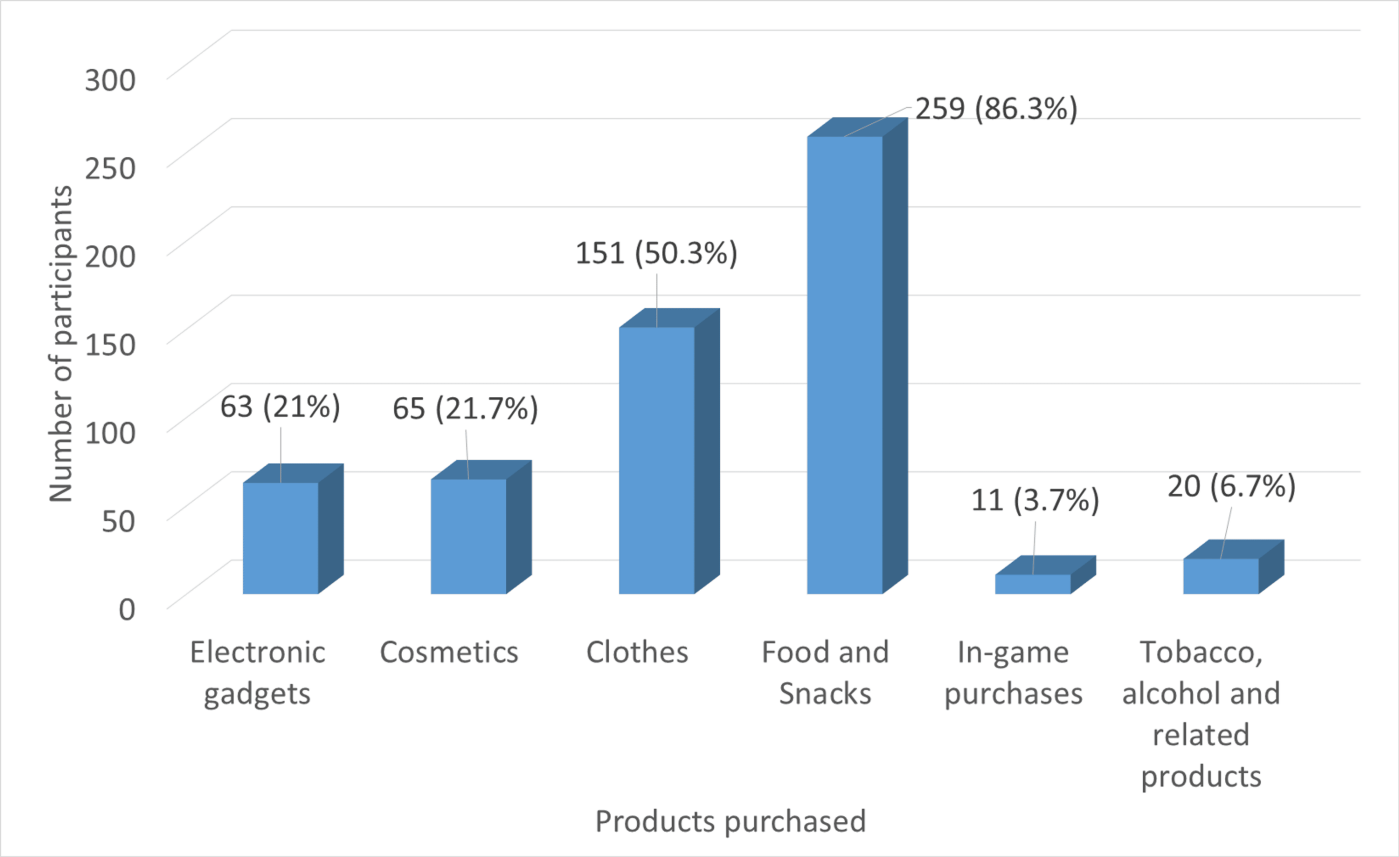
Expenditure pattern

Figure 1 shows where the study participants spend more money. Participants could choose multiple options if they found more than one option suitable. Most of them (259 (86.3%)) spent a lot on food and snacks, followed by clothes, on which around half of them (151 (50.3%)) spent a great deal of money. Approximately one-fifth of students spent a fair amount of money on electronic gadgets (63 (21%)) and cosmetics (65 (21.7%)). 20 (6.7%) of the participants spent more money on tobacco, alcohol, and related products and 11 (3.7%) of them on in-game purchases.

**Figure 2:**
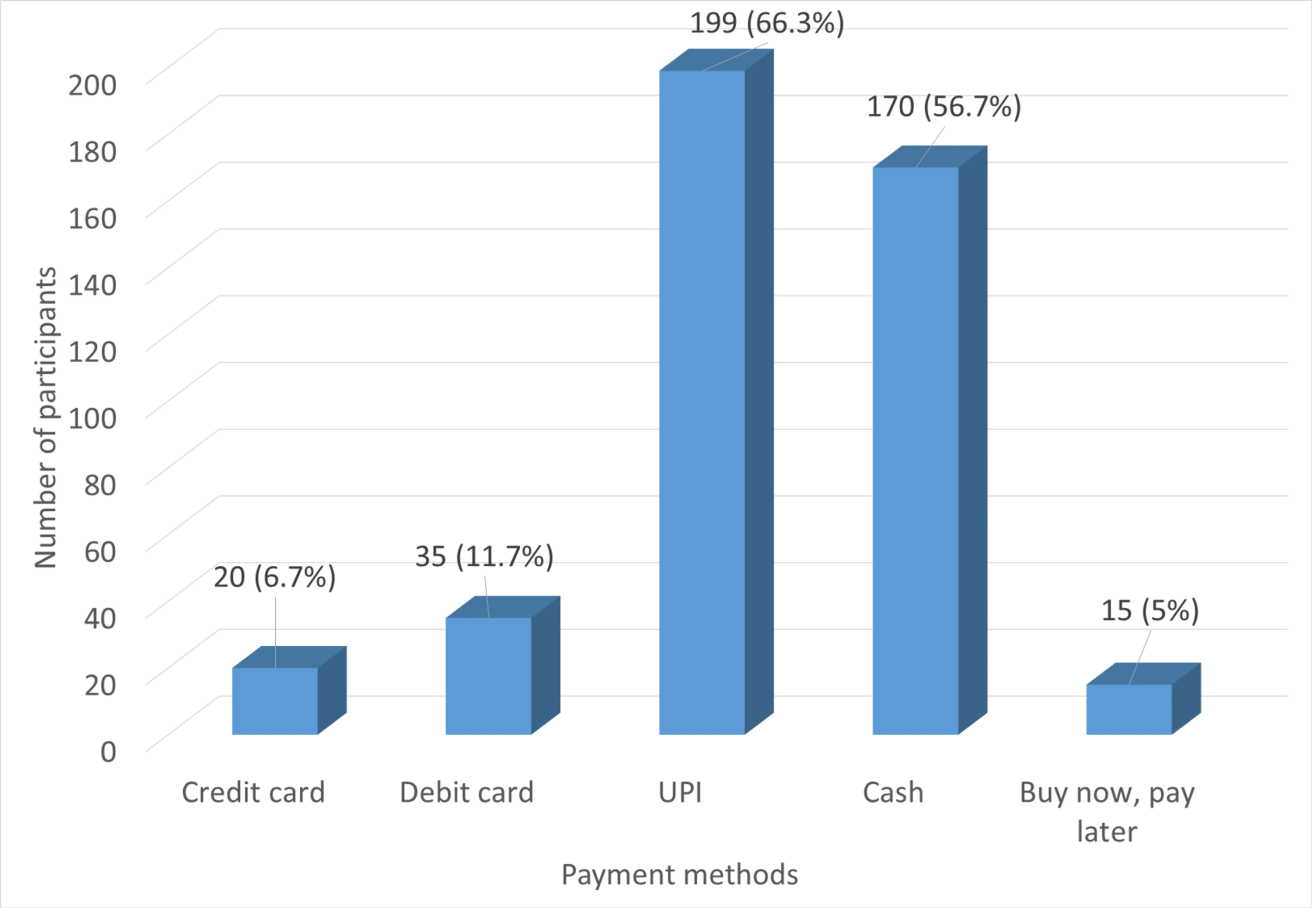
Preferred mode of payment UPI: Unified Payments Interface

Figure 2 shows the participants’ preferred mode of payment, and they were free to choose more than one option if they found it appropriate. 199 (66.3%) of the students preferred Unified Payments Interface (UPI) payment, followed by cash, which was preferred by a little over half (170 (56.7%)) of the study participants. The payment modes that were liked better by the least number of people were ‘Buy Now, Pay Later’ (15 (5%)) and credit card (20 (6.7%)).

The bivariate analysis of the study variables and the outcome, CBD, is given in Table 3. It was found that students who had their own source of income had 3.689 times higher odds of having CBD when compared to those who did not, with p values of 0.012 and 95% CI = 1.246-10.918. Participants whose spending habits were influenced by social media had 3.647 times more odds of having CBD than those who were not influenced by social media. Other variables that were found to be significantly associated with CBD were living away from parents, being tempted to buy during offers and deals, being influenced by social circles, and not having a habit of saving money.

**Table 3:** Bivariate analysis of compulsive buying disorder and its related variables * p-value <0.05, statistically significant at 95% confidence interval † 95% confidence interval

S. No.	Variables	Category	Has compulsive buying disorder		Doesn’t have compulsive buying disorder		Unadjusted odd’s ratio (95% CI) †	p-value
-	-	-	Frequency (n)	Percentage (%)	Frequency (n)	Percentage (%)	-	-
1.	Age	17-20	33	25	99	75	1.267 (0.737-2.178)	0.392
		21-24	35	20.8	133	79.2		
2.	Gender	Female	42	21.3	155	78.7	0.802 (0.458-1.405)	0.441
		Male	26	25.2	77	74.8		
3.	Year of study	1st and 2nd year	38	23.2	126	76.8	1.066 (0.619-1.836)	0.819
		3rd and 4th year	30	22.1	106	77.9		
4.	Residence	Living away from parents	40	28.8	99	71.2	1.919 (1.109-3.322)	0.019*
		Living with parents	28	17.4	133	82.6		
5.	Own source of income	Yes	7	50	7	50	3.689 (1.246-10.918)	0.012*
		No	61	21.3	225	78.7		
6.	Pocket money per month	More than Rs. 5000	28	28.6	70	71.4	1.620 (0.927-2.832)	0.089
		Less than Rs. 5000	40	19.8	162	80.2		
7.	Accountability to parents	Not accountable	18	25.7	52	74.3	1.246 (0.670-2.318)	0.487
		Accountable	50	21.7	180	78.3		
8.	Financial support from others	Supported by others	14	22.6	48	77.4	0.994 (0.509-1.939)	0.986
		Not supported by others	54	22.7	184	77.3		
9.	Has savings or recurrent deposit account	Doesn't have a savings account	34	22.8	115	77.2	1.017 (0.593-1.747)	0.950
		Has savings account	34	22.5	117	77.5		
10.	Tempted to buy during offers and deals	Yes	46	34.1	89	65.9	3.360 (1.895-5.957)	0.000*
		No	22	13.3	143	86.7		
11.	Ever been scolded by parents for spending too much	Yes	40	34.2	77	65.8	2.876 (1.651-5.009)	0.000*
		No	28	15.3	155	84.7		
12.	Spending influence by social circle	Yes	34	33.3	68	66.7	2.412 (1.387-4.193)	0.002*
		No	34	17.2	164	82.8		
13.	Social media influence on spending	Yes	45	35.7	81	64.3	3.647 (2.062-6.451)	0.000*
		No	23	13.2	151	86.8		
14.	Habit of saving money	No	25	36.8	43	63.2	2.555 (1.411-4.628)	0.002*
		Yes	43	18.5	189	81.5		
15.	Been taught about financial saving	Yes	22	22	78	78	0.944 (0.531-1.681)	0.845
		No	46	23	154	77		

The logistic regression of variables found to be significant in the bivariate analysis is shown in Table 4 with the adjusted odds ratio. Not having a habit of saving money was found to be significant with an adjusted odds ratio of 3.115 for people who saved money (p-value 0.002 and CI=1.501-6.463). Other variables found to be significantly associated with CBD are being tempted to buy during offers and deals, and spending is influenced by social media.

**Table 4:** Logistic regression between compulsive buying disorder and its related variables * p-value <0.05, statistically significant at 95% confidence interval † 95% confidence interval

S. No.	Variables	Category	Unadjusted odd’s ratio (95% CI) †	p-value	Adjusted odd’s ratio (95% CI) †	p-value
1.	Residence	Living away from parents	1.919 (1.109-3.322)	0.019	1.503 (0.794-2.844)	0.210
		Living with parents				
2.	Own source of income	Yes	3.689 (1.246-10.918)	0.012	2.735 (0.778-9.621)	0.117
		No				
3.	Tempted to buy during offers and deals	Yes	3.360 (1.895-5.957)	0.000	2.728 (1.385-5.372)	0.004*
		No				
4.	Ever been scolded by parents for spending too much	Yes	2.876 (1.651-5.009)	0.000	1.457 (0.770-2.758)	0.247
		No				
5.	Spending influence by social circle	Yes	2.412 (1.387-4.193)	0.002	1.192 (0.586-2.424)	0.628
		No				
6.	Social media influence on spending	Yes	3.647 (2.062-6.451)	0.000	2.127 (1.053-4.299)	0.036*
		No				
7.	Habit of saving money	No	2.555 (1.411-4.628)	0.002	3.115 (1.501-6.463)	0.002*
		Yes				

## Discussion

CBD is one of the underreported conditions that, if left unchecked, could lead to financial problems, and the person affected could end up with mental health problems such as depression and anxiety. The results of the present study, which was performed to determine the prevalence of CBD, compared with those of studies conducted elsewhere, are discussed below. 

In the present study, the prevalence of CBD was 22.6%. According to Black et al., the lifetime prevalence of CBD is 5.8% in the US [15]. A study conducted by Amin et al. in Saudi Arabia and Narayanan et al. in Chengalpattu district found the prevalence of CBD to be 18.2% and 29.7%, respectively [16,17]. A study by Lejoyeux et al. in France reported the prevalence of CBD to be 11% [18]. A comparatively lower prevalence of 8.3% was reported in a study by Maraz et al. in England [19]. As the prevalence was found to be as low as 8.3% to almost one-fourth of participants being affected in some studies, it could be attributed to various factors such as demographics, economic status of the population, and the data collection tool used to quantify CBD.

According to the present study, males were more commonly affected (26 (25.2%)) than female participants (42 (21.3%)). Contradictory findings were found in studies conducted by McElroy et al. and Schlosser et al. in the US, in which women were more commonly affected than men based on the notion that women love shopping and indulge in that activity more than men [7,8]. These findings could be further explored to identify spending patterns by gender through qualitative interviews [8].

In this study, those who were tempted to buy during offers and deals were found to have a statistically significant association with CBD. A motivation theory put forth by Kukar-Kinney et al. found that high-pressure indicators in the form of daily deals and offers affect the vulnerable population, leading to CBD and overspending [20]. Reducing these advertisements was found to have a positive impact on the spending behavior of the study population, which could in turn reduce the CBD [20].

According to the present study, participants who were influenced by social media tended to have a higher prevalence of CBD. A study done by Park and Chun found that luxury images and consumer items used by social media influencers could stimulate luxury purchases among the youth population, which could in turn lead to compulsive buying [21]. A study done by Alajlan and Saleh in Saudi Arabia found that materialism mediates the relationship between advertisements on social media and compulsive buying behavior [22]. A study by Singh et al. found that fear of missing out (FOMO) due to exposure to goods and products through social media can trigger CBD among individuals [23].

CBD was found to be more common among those who did not have the habit of saving money. Poor management of financial resources is a common problem, especially among college students [24]. When exposed to situations in which they have to maintain their social image or status, they tend to impulsively spend money on maintaining their status quo among their peers. This could, in turn, lead to CBD and guilt and self-sabotage, especially when they end up with financial problems. Similar findings were observed in a study done by Harnish et al. [25].

The major strength of the study lies in the fact that the study population is medical students, who are already subjected to high academic pressure and various sociocultural pressures that predispose them to these behavior patterns. The use of a standardized, validated questionnaire enhanced the reliability of the findings. The cross-sectional nature of this study is a major limitation, as it is difficult to establish a causal relationship.

## Conclusions

CBD is an often-overlooked behavioral concern in academic settings, and its presence is highlighted in this study. These findings underscore the influence of social media, peers, and strategic deals by e-commerce services on students’ purchasing habits. It is important to identify and treat CBD in the early stages to prevent long-term financial and psychological consequences. Educational institutions should integrate awareness programs and mental health support systems. Parents should be counseled to prevent CBD in children. Further research is needed to explore the longitudinal patterns and associated risk factors. Addressing this issue holistically can contribute to the overall well-being of future healthcare professionals.

## Appendices

Appendix A

**Table 5:** Questionnaire to collect socio-demographic details and information about variables related to compulsive buying disorder UPI: Unified Payments Interface

S. No.	Variables	Answers
1.	Participant ID	
2.	Age	
3.	Gender	
	Male	
	Female	
	Other	
4.	Year of study	
5.	Residence	
	Living away from parents	
	Living with parents	
6.	Do you have your own income source?	
	Yes	
	No	
7.	How much pocket money do you receive per month?	
8.	Are you supported financially by people other than your parents?	
	Yes	
	No	
9.	Are you accountable to your parents for your spending?	
	No	
	Yes	
10.	Do you have a savings or recurrent deposit account?	
	No	
	Yes	
11.	Are you tempted to buy during offers and deals?	
	Yes	
	No	
12.	Have you ever been scolded by your parents for spending too much money?	
	Yes	
	No	
13.	Do you feel that your spending behaviour is influenced by social circle (other than social media)?	
	Yes	
	No	
14.	Does social media play a role in your buying behaviour?	
	Yes	
	No	
15.	Do you have a habit of saving money?	
	Yes	
	No	
16.	Did any in your family or school teach you about financial savings?	
	Yes	
	No	
17.	What are the things you spend money the most on?	
	Electronic gadgets	
	Cosmetics	
	Clothes	
	Food and snacks	
	In-game purchases	
	Tobacco, alcohol and related products	
18.	What is your preferred mode of payment?	
	Credit card	
	Debit card	
	UPI	
	Cash	
	Buy now, pay later	

Appendix B

**Table 6:** Richmond Compulsive Buying Scale Source: Ridgway et al. (2008) [13]

	Strongly Disagree						Strongly Agree
	1	2	3	4	5	6	7
1) My closet has unopened shopping bags in it.							
2) Others might consider me a schopaholic.							
3) Much of my life centers around buying things.							
4) I consider myself an impulsive purchaser.							
	Never						Very Often
	1	2	3	4	5	6	7
5) I buy things that I don’t need.							
6) I buy things I did not plan to buy.							
